# Practical Aspects of Medical Science

**Published:** 1894-02-17

**Authors:** 


### Practical Aspects of Medical Science.

A very interesting discussion took place recently at the New York Academy of Medicine on the "Relative Value of the Important Factors of Longevity." Besides its own intrinsic interest, the subject was yet further enlivened by being treated from the two-fold point of view of the general practitioner and the examiner of life insurance companies, each bringing their own experience to bear on the question. It is not given to any of us to know the length of our days or the number of our years, but there are certain general principles deduced from actual facts which have now resolved themselves into accepted theories regarding the span of human life. Everyone who has given the subject something more than a passing consideration agrees in admitting that the principal factors which, *par excellence*, make for longevity are, firstly, hereditary ones; and secondly, personal. Even in the circumscribed limits of our individual acquaintance it has been forced upon us how important for length of life is the inheritance from long-lived ancestry. Like begets like in no case more strongly than in this. Dr. M. Morris, on the occasion of the discussion at New York to which we refer, among many pertinent remarks on this subject, said: "There was an indefinable something in the human organism, varying in degree and force, termed tenacity of life or natural resistance to disease, by which some persons passed most successfully through serious maladies and injuries, whilst others without so much natural resistance succumbed." On the same authority we have it that among the greater proportion of those persons who are endowed with this "tenacity of life" their ancestry has been found to be long lived. "It is not, of course, in human ken," he goes on to say, "to describe this vital principle, but by observation it was known that certain conditions would cut it short, yet it was impossible to prolong it by any means whatever beyond the natural inheritance. Some men are at best endowed with but a short life, whilst others, if obedience is paid to natural laws, have the promise of a long span of life in them." It is not given to us in the ordinary course of events to say this man will live long—that one will die young—at best we can form conclusions, based on observation and experience of what the probability of existence are. So much has to be taken into account, personal and exterior conditions, which all have a direct bearing on the question of probable longevity. Habits of living, occupation, observance of sanitary laws and residence, all necessarily exert an influence, varying as they themselves vary. Life-shortening influences resultant on acute diseases, bad habits, excessive indulgences, unfavourable residence, and the like, must all be taken into account. Yet in all kinds of life, as Dr. Morris says, we must find some substance in common in which life inhered, and upon which life must depend. Plant life, for instance, presents forms of structure to us which cannot be found in animal life, whilst in the latter, again, structures are traceable which are confined to that particular kingdom. But there is one substance common to all living things, one substance in which all forms of life alike present uniformity, and that is the secretory tissue with which they are endowed. "This also," our authority observes, "has its lifetime, and when it ceases to exist life becomes extinct. This secretory tissue is the only kind of substance which is transmitted from the ancestors, and therefore it contains in itself all the ancestral influences which are transmitted. But there are different kinds of secretory tissue, and different proportions of each, so that the duration of life is, after all, but a variable quantity. It is not unusual to remark," Dr. Morris goes on to say, "that a person inherited red hair from the mother, or a dark complexion from the father, and so on; why, then, not inherit the stomach from one parent, the liver from another, and similarly with other organs?" The different organs of the body are not to be judged as units, but as aggregations; and why should resemblance be limited to exterior characteristics? If it were so without, why not within? One might fairly reason that the similitude does not end with the external form. Dr. Morris illustrated his question. "If," he asked, "a great grandfather died of heart disease at 71, a father at 65, might not a son resembling this father rightly infer that he would die still younger of the same disease? But instead of calling it disease, call it natural life shortened by a weakness in the paternal ancestry."

Another participant in this discussion, Dr. E. W. Lambert, whilst agreeing in the main with the substance of the above, was more inclined in considering this subject to give greater weight to the environment, temperament, and manner of living of the individual than to lay entire stress on what he had inherited from his forefathers. "The man," he says, "who was careful, considerate, and moderate in the exercise of his faculties, whether animal or intellectual, was one who would last longer than the man who over-indulged in any one of the numerous things which go to make up life. The men who broke down prematurely were usually those who had not lived temperately."

Quite so, but granted two men with parallel conditions in temperament and in surroundings, the one coming of a long-lived stock, the other not, apart from their habits, is not the advantage in favour of longevity more on the side of the one whose family history evinces that power of resistance called long living?

There is another important factor also of longevity, and that is the actual bodily form of the person; indeed, this condition ranks only second to inherited constitution in the opinion of one authority on the subject.

Indeed, from the point of view of an insurance company (and is there a more crucial test?), the farther a man is away from the ordinary standard of build, the more critically has his family history to be inquired into. Extremes in weight, either above or below the average, are both alike regarded critically by such companies.

Mistakes and mistaken calculations of course have necessarily been made by those most versed in speculations of the probable span of human existence, the solving of which problem is not granted to man's intelligence. At best we can put together the results of our experience, and from these results establish certain general principles, which we call theories.

